# GNINA 1.3: the next increment in molecular docking with deep learning

**DOI:** 10.1186/s13321-025-00973-x

**Published:** 2025-03-02

**Authors:** Andrew T. McNutt, Yanjing Li, Rocco Meli, Rishal Aggarwal, David Ryan Koes

**Affiliations:** 1 Department of Computational and Systems Biology, University of Pittsburgh, Pittsburgh, PA USA; 2 Computational Biology Department, Carnegie Mellon University, Pittsburgh, PA USA; 3 Department of Biochemistry, University of Oxford, Oxford, OX1 3QU UK; 4 Present Address: Swiss National Supercomputing Center (CSCS), ETH Zurich, 6900 Lugano, Switzerland

**Keywords:** Molecular docking, Deep learning, Structure-based drug design

## Abstract

Computer-aided drug design has the potential to significantly reduce the astronomical costs of drug development, and molecular docking plays a prominent role in this process. Molecular docking is an *in silico* technique that predicts the bound 3D conformations of two molecules, a necessary step for other structure-based methods. Here, we describe version 1.3 of the open-source molecular docking software Gnina. This release updates the underlying deep learning framework to PyTorch, resulting in more computationally efficient docking and paving the way for seamless integration of other deep learning methods into the docking pipeline. We retrained our CNN scoring functions on the updated CrossDocked2020 v1.3 dataset and introduce knowledge-distilled CNN scoring functions to facilitate high-throughput virtual screening with Gnina. Furthermore, we add functionality for covalent docking, where an atom of the ligand is covalently bound to an atom of the receptor. This update expands the scope of docking with Gnina and further positions Gnina as a user-friendly, open-source molecular docking framework. Gnina is available at https://github.com/gnina/gnina.

**Scientific contributions**: GNINA 1.3 is an open source a molecular docking tool with enhanced support for covalent docking and updated deep learning models for more effective docking and screening.

## Introduction

The development of new drugs is a complex and time-consuming process [27], requiring the evaluation of large numbers of compounds to identify those with therapeutic potential. Molecular docking, a *in silico* technique that models the 3D binding conformation of small molecules to proteins, is a key tool for accelerating this process [22]. Predicting the binding conformation of small molecules to their target proteins enables prioritization of compounds for experimental testing along with enabling other *in silico*, structure-based methods such as lead optimization and binding affinity prediction.

One widely used, open-source molecular docking pipeline is Gnina [18], a fork of Autodock Vina [33] and Smina [13]. The docking workflow follows a conventional setup, where ligand conformational sampling is carried out via a set of Markov chain Monte Carlo (MCMC) chains that randomly perturb the ligand in the specified binding site. Following sampling, protein-ligand conformations are scored and ranked with the top poses output to the user. Gnina distinguishes itself from its predecessors by using convolutional neural network (CNN) scoring functions that work on an atomic density grid representation (i.e., a 3D “picture” of the complex) within the docking workflow [25]. The ligand poses from the MCMC chains are first minimized with respect to the Autodock Vina scoring function, and then rescored and ranked using the CNN scoring functions. An ensemble of CNN scoring functions of differing computational complexity is used to score the ligand poses, which enhances the binding pose prediction at the cost of additional computation. Gnina has performed well in prospective applications [14] and independent evaluations consistently find it outperforms Vina and achieves similar performance to commercial tools [7]. Recent works have also shown that the performance of GNINA can be further boosted through the use of multiple conformers of the small molecule [19].

We present incremental improvements to the docking pipeline resulting in Gnina 1.3. These changes include the introduction of covalent docking capabilities, retraining of the CNN scoring function on updated datasets for higher quality models, and the development of knowledge distilled CNN scoring functions for faster scoring. Furthermore, we establish Gnina as a platform to enable deep learning development in docking by integrating PyTorch as the supported deep learning framework. These enhancements expand the scope, accuracy, and computational efficiency of Gnina, further solidifying its position as a valuable, open-source tool in the pursuit of computationally developed therapeutics.

## Implementation

### Caffe replaced with PyTorch

Gnina 1.0 uses the venerable Caffe [12] C++ deep learning framework to implement its convolutional neural network scoring. Since the initial development of Gnina’s CNN scoring model [25], more flexible, powerful, and popular deep learning frameworks have been released. Specifically, the PyTorch [23] framework has come to dominate the deep learning community, with more than 90% of models on the popular HuggingFace model sharing site being PyTorch exclusive. PyTorch, and the underlying PyTorch C++ backend, supports a robust ecosystem of developers and users and provides a flexible, auto-differentiation based approach that enables rapid prototyping and the development of sophisticated model architectures. With Gnina 1.3, Caffe has been replaced with PyTorch. This introduces no changes to typical usage, but makes it easier for advanced users to integrate their own PyTorch trained models into a conventional docking workflow and sets the stage for more substantive changes in future Gnina releases, such as augmenting the Monte Carlo sampling with deep neural network directed sampling [4, 7].

### Retrained models

The CrossDocked2020 dataset [9] used for training of the Gnina CNN scoring functions has been updated to version 1.3 since the initial Gnina 1.0 models were trained. The updated version 1.3 addresses ligand and receptor misalignment problems and incorrect bond typing problems present in earlier versions (statistics of the updated datasets are provided in Table S1 and Figure S1). All models trained on CrossDocked2020 or ReDocked2020, a redocked-only subset of CrossDocked2020, [9] were retrained on the updated version of their corresponding dataset. Models input a 3D grid of Gaussian atom-type densities generated by the libmolgrid library [30]. All models are trained for two tasks: pose scoring and binding affinity prediction. The pose score is trained to classify if a pose is $\le$ 2Å RMSD from the ground truth using a cross entropy loss function. The binding affinity is trained with a mean squared error loss between the predicted and ground-truth affinity that is hinged if the pose is inaccurate. Further training details and hyperparameters are provided in the supplement.

After retraining the models, we greedily selected an ensemble of models with the best performance on both the redocking and cross-docking tasks following the Default Ensemble selection procedure enumerated in McNutt et al. [18]. This results in an ensemble of three models compared to the default Gnina 1.0 ensemble, which has five models.

### Knowledge distillation for faster screening

McNutt et al. [18] found that ensembles of CNN scoring functions always produced higher quality docked poses than a single CNN scoring function when used in the Gnina docking pipeline. However, utilizing an ensemble of CNN scoring functions incurs a greater computational cost than using a single CNN scoring function. This extra computational burden is especially egregious when running Gnina without a GPU (458 s and 72 s for the best ensemble and single model, respectively in Gnina 1.0), a common scenario when utilizing Gnina for high throughput screening. Knowledge Distillation (KD) is a technique to condense the knowledge of a large “teacher” model into a smaller “student” model, enabling faster inference with similar model performance [10]. Ensemble KD transfers the knowledge learned by multiple teacher models to a single student model by minimizing the discrepancy between the average representation of the teachers and the student [2, 32]. Ensemble KD can reduce the computational overhead of workflows that use an ensemble of large models without significantly impacting performance.

**Fig. 1:**
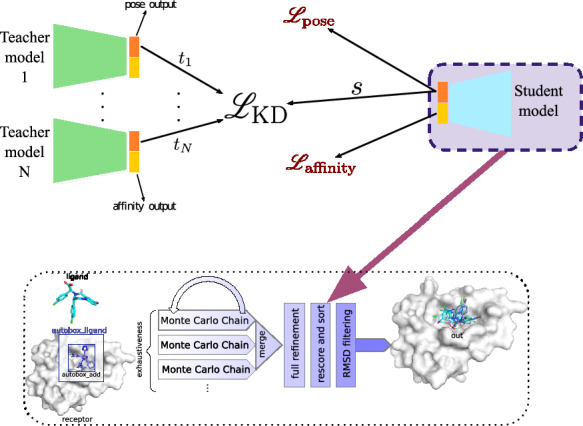
Knowledge distillation condenses the pose scoring power of the teacher ensemble into a single student model. The student model is trained to reproduce the pre-softmax pose score logits of the ensemble of teacher models and simultaneously trained on the ground truth pose and affinity labels. The student model is then used to rescore and rank poses in the Gnina docking pipeline to speed up docking

There are four different CNN models for molecular docking within Gnina that differ in their model architecture and training set [18]. The two architectures are “Default 2018”, a linear CNN with five convolutional layers, and “Dense”, which has twelve convolutional layers organized into three densely connected blocks [11]. In addition to the full CrossDocked2020 dataset, models are also trained on a subset that consists of only redocked poses: ReDocked2020 [9]. Each CNN model has five variants that only differ in their training initialization (random seed). These five variants form an ensemble for each CNN model. We utilize ensemble KD to compress the ranking performance of the ensemble of five variants into a single student model with the same architecture (Fig. 1). Additionally, we consider one more ensemble of the CNN models: “All Default2018 Ensemble”, consisting of all CNN models with the Default2018 architecture. Default Gnina docking only utilizes the pose score of the CNN models, therefore our distillation only considers the pose score with our KD loss being the sum of Kullback–Leibler (KL) divergence of the pre-softmax values of the pose score between the student and each teacher. The total training loss is a sum of the KD loss and the ground truth affinity and pose classification losses. Training is carried out on the same training dataset as the teachers. For the “All Default2018 Ensemble”, we train the student on the CrossDocked2020 v1.3 dataset since this is largely a superset of the training datasets used for the Default2018 models (CrossDocked2020, ReDocked2020, and PDBBind General v2016). This leads to the creation of 6 CNN scoring functions distilled from ensembles.

More details about the training and hyperparameters of the ensemble KD can be found in McNutt et al. [17].

### Covalent docking

**Fig. 2:**
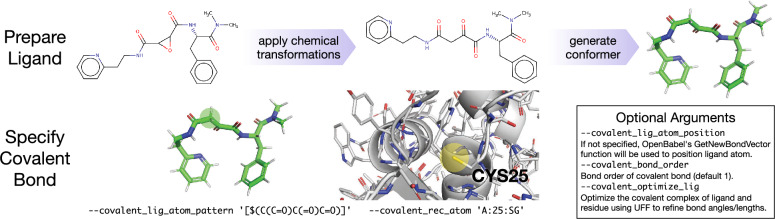
Covalent docking with Gnina. The input ligand must be provided as conformation representative of the bound form the ligand, including any chemical modifications (e.g. epoxide ring opening). The covalent atom on the ligand is specified with a SMARTS expression; all matching atoms are evaluated. The covalent atom on the receptor is specified with the chain identifier, residue number, and atom name. Additional optional arguments refine the positions and treatment of the covalent bond

Gnina 1.3 provides a simple interface for covalent docking, as shown in Fig. 2. Instead of presuming a particular chemical reaction, Gnina expects the bound, covalent form of the ligand to be provided as input (as is the case with other programs [1, 3, 15, 34, 35]). The user then specifies the ligand atom, using a SMARTS expression, and a receptor atom, using the chain, residue ID, and atom name. If multiple ligand atoms match the SMARTS expression, all pairings of ligand and receptor atoms are evaluated, resulting in a corresponding expansion of the number of output poses. Given a pairing of receptor and ligand atoms, the ligand is re-positioned so that the ligand atom is within bonding distance of the receptor atom, the bond is created with a user configurable bond order (default of one), and the residue-ligand construct is treated as one flexible residue while docking. That is, the internal torsion angles are sampled and optimized during Monte Carlo sampling and energy minimization, but no rigid body transformations are performed. For purposes of CNN scoring, which treats receptor and ligand atoms as having different types, the ligand atoms remain identified as ligand atoms. In order to position the ligand at a reasonable location, by default the OpenBabel [21] GetNewBondVector heuristic is applied to the receptor atom (after reducing the number of hydrogens) to identify a logical placement of the ligand covalent atom. Alternatively, this position can be manually specified. The OpenBabel method OBBuilder::Connect is then used rotate and translate the ligand such that the covalent ligand atom is positioned appropriately and the bonding geometry is reasonable. Optionally, the entire residue-ligand construct can be optimized using the UFF force field to further refine the bonding geometry.

## Results

We enumerate the improved performance, both in terms of run-time, cross-docking pose prediction accuracy, and virtual screening of Gnina 1.3.

Docking runtime is reported as the average time to dock a protein-ligand complex, computed over a random 100 complex subset of the PDBbind core set (further detailed in the supplement). Pose prediction accuracy is measured via TopN, defined as the percentage of protein-ligand complexes where a $\le 2$ Å RMSD pose is found within the top N ranked poses. Virtual screening metrics are described in Sect. Virtual Screening.

**Fig. 3:**
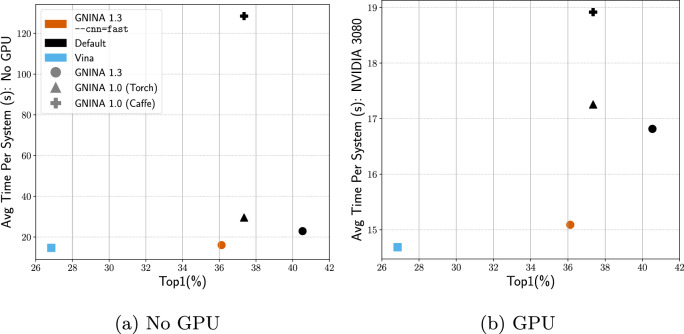
Comparing cross-docking Top1 and the computational cost of utilizing Gnina’s CNN scoring functions for docking, both with and without a GPU (note that the y-axis has different scales). Both the 1.3 Default Ensemble and the fast model sit on the Pareto-frontier of the docking accuracy and computational cost curve. Results for redocking performance are provided in Figure S6 and Table S7

### Torch performance

Docking is often used for virtual screening of large libraries, which requires a scoring function that is fast without compromising accuracy. We benchmark the Gnina CNN models on a random 100 complex subset of the PDBbind core set v.2016 [29] to determine their computational cost (details of the benchmarking can be found in the supplement). Replacing the Caffe models with a PyTorch implementation of the same models produces no change in pose performance, but does result in a significant run-time performance improvement in CPU-only mode as shown in Fig. 3. Average docking time reduces from 129 s to about 30 s per complex when no GPU is used during docking. This is in part due to better support for multi-processing in PyTorch. For our benchmarking we limited Gnina to using four cores, therefore the performance benefit is potentially even greater than shown in Fig. 3 for many-core systems (Figure S2).

**Fig. 4:**
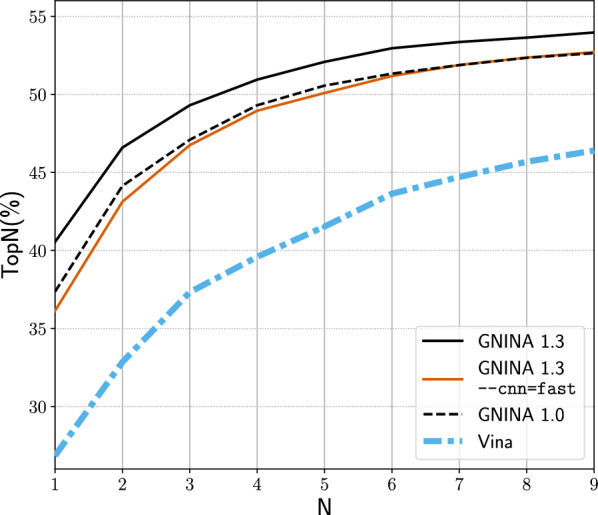
Cross-docking performance of the GNINA scoring functions on the Wierbowski et al. [36] dataset

### Updated models

We consider the performance of our updated models both at pose prediction and virtual screening.

#### Pose prediction

We consider two tasks: redocking and cross-docking. Redocking, removing a ligand from a complex structure and docking it back in place, provides an easily verifiable benchmark for molecular docking methods, while cross-docking represents a realistic use case of molecular docking: docking a ligand to a non-cognate receptor. For the cross-docking evaluations, we utilize the Wierbowski et al. [36] cross-docking dataset. The redocking evaluations utilize the Posebusters benchmark set and the Astex diverse set as defined in Buttenschoen et al. [5]. Further dataset information is provided in Table S4. We find that all of the retrained models rank poses more accurately when cross-docking, but the retrained redock_default2018 models are about the same at pose ranking for redocking (Figure S3 and S4). These improvements are due to the updated CrossDocked2020 dataset. We see additional improvements through ensemble knowledge distillation; while the distilled models are not as good as the full ensemble, they are better than any single un-distilled model (Table S5 and S6).

The updated default ensemble is composed of a retrained dense model, a knowledge distilled dense model, and a knowledge distilled crossdock_default2018 model (all models are trained on the full CrossDocked data set). We see in Fig. 4 that the new Gnina 1.3 Default Ensemble ranks cross-docked poses better than the 1.0 Default Ensemble for all N, increasing Top1 from 37% to 40%, and is faster with an average CPU-only time of 23 s compared to 30 s using the 1.0 Default Ensemble. However, redocking Top1 drops slightly on both datasets (Figure S5), decreasing from 69% to 67% on the Posebusters Benchmark set.

A new feature in Gnina 1.3 is a “fast” single model, the best performing Default2018 model. This model was distilled from the “All Default2018 Ensemble”, which consists of all models trained using this architecture. This model is enabled with the command-line option –cnn=fast and is intended to be used during high-throughput screening. As shown in Fig. 3, the fast model has only slightly decreased TopN compared to the 1.0 Default Ensemble when cross-docking, but is significantly faster with an average CPU-only time of 16 s, only 1.3s slower than using the Vina empirical scoring function and less than 1 s slower than when using a GPU (Table S7). We see a larger gap in performance between the 1.0 Default Ensemble and the fast model on redocking (Top1 of 69% and 64% for the 1.0 and fast model, respectively).

**Fig. 5:**
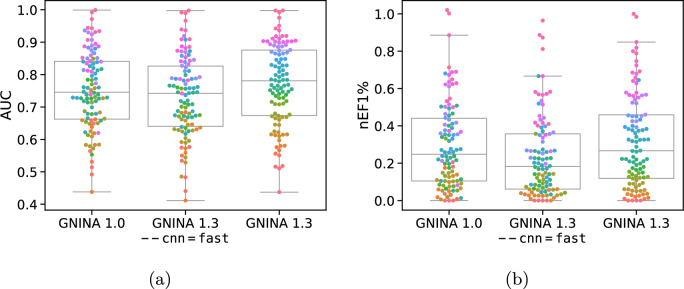
Virtual screening results on DUD-E for GNINA 1.3 compared with GNINA 1.0. Both the default scoring and the “fast” option are evaluated using (**a**) area under the ROC curve (AUC) and (**b**) normalized enrichment factor of the top 1%. Each data point corresponds to the performance of a specific, uniquely colored, DUD-E target

#### Virtual screening

Retrospective virtual screening results for Gnina 1.3 on the DUD-E [20] benchmark are shown in Fig. 5. Compounds are ranked using the pose score (CNNscore). We note that while there are known biases in the DUD-E benchmark that complicate evaluation of machine learned scoring function [6, 28], Gnina was not trained on DUD-E data and so is not directly effected by these biases. Both the area under the receiver operating characteristic (AUC) and the enrichment factor [24] at 1% (EF1%) are reported. EF1% measures the ratio of active compounds ranked in the top-1% of a virtual screen to a random selection of the database with the same size. As the enrichment factor is sensitive to class imbalances, we normalize by the best possible EF1% so the metric (denoted nEF1%) is comparable across targets [31]. Gnina 1.3 generally outperforms 1.0, with a median AUC and nEF1% of 0.78 and 0.27 compared to 0.75 and 0.25 for Gnina 1.0. Gnina 1.3 improves upon 1.0 for 68 of the 102 targets. The single, ‘fast’ 1.3 model has comparable AUCs to 1.0, but worse enrichment factors.

**Fig. 6:**
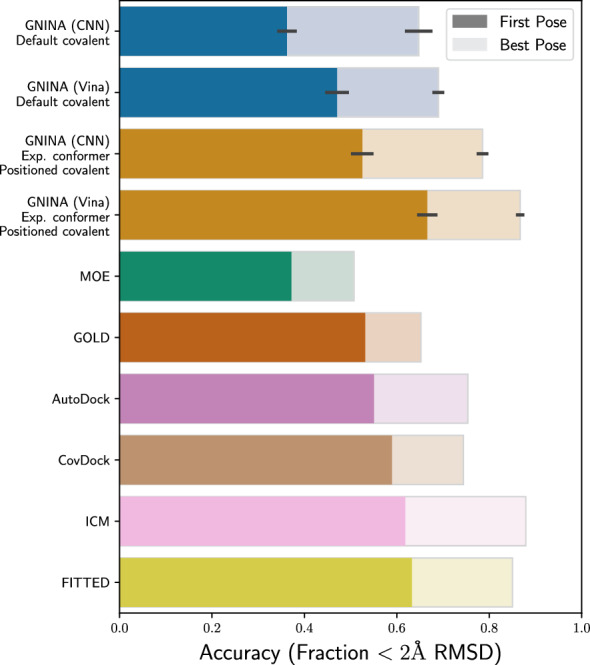
Gnina covalent docking performance in terms of fraction of targets where the top ranked pose (darker shade) or any sampled pose (lighter shade) is within 2Å RMSD of the experimental structure. Error bars display the standard deviation across five docking runs initialized with different random seeds. Accuracy of other approaches is sourced from Scarpino et al. [26]

### Covalent docking

To evaluate the new covalent docking feature in Gnina 1.3, we use a benchmark of 207 complexes from Scarpino et al. [26]. Use of this covalent redocking benchmark allows us to compare to previously evaluated approaches in Fig. 6. We consider two scenarios: default covalent docking where a generated conformer of the ligand is used with no additional positioning information, and docking the experimental conformer with a precisely specific location of the covalent ligand atom. This provides the expected range of performance depending on the amount of prior information available; results for in-between settings can be found in Figure S7. The success rate for Gnina ranges from the worst (36.2%) to the best (66.6%) depending on the settings used. Using the Vina scoring function results in significantly better performance than the CNN. This is unsurprising, as the CNN was not trained on any covalent complexes, and points to a common pitfall of applying models outside their domain of applicability. Using CNN scoring on this same benchmark but without covalent docking does outperform Vina scoring, with a 27.5% success rate compared to Vina’s 15.8% (both of which are significantly worse than enabling covalent docking). Overall, when using Vina scoring (–cnn_scoring=none), covalent docking with Gnina 1.3 is competitive with, but does not outperform, the state of the art.

## Discussion

We present Gnina 1.3, an incremental improvement to the original Gnina software that lays the groundwork for more substantive future changes. Gnina now utilizes the PyTorch deep learning framework instead of Caffe, which allows quicker and easier integration with novel deep learning methods. Additionally, the switch to PyTorch reduces the computational cost of using the CNN scoring functions as shown in Fig. 3 and Table S7.

The built-in CNN scoring functions have been retrained on the most up-to-date version of the CrossDocked2020 dataset, which has increased the ranking performance on the cross-docking task. We find the retrained models show slightly reduced performance on redocking (Figures S5, S4), however, the CNN scoring functions still show superior ranking power to the Vina scoring function. The reduction in redocking performance is likely due to a reduction in the number of redocked poses in the CrossDocked2020 v1.3 dataset through filtering of problematic poses. Redocking is largely a synthetic benchmark for molecular docking as prospective drug discovery requires docking a ligand into a non-cognate receptor, so prioritizing improvements in cross-docking performance is a sensible strategy.

Finally, we utilized KD to reduce the computational burden of the highest performing CNN scoring functions without significantly reducing the pose ranking power of the models. Condensing CNN ensembles into a single model, in addition to the move to PyTorch, now enables an increase in Top1 cross-docking relative to Vina from about 25% to 36% with only a 1.5 s increase in average docking time without using a GPU. This will allow for much faster and cheaper screening of ultra-large libraries for drug discovery campaigns, like that in Li et al. [14] which docked 7 million compounds. Additionally, we now provide the option –cnn fast for high-throughput screening. This option is most appropriate for running many single-threaded docking jobs that will be followed by a rescreen of the top hits using the v1.3 Default ensemble to reduce the number of false positives. When ample compute or GPUs are available, the run-time performance improvement of this single fast model is likely not sufficient to justify a hierarchical screening strategy.

Due to the integration of PyTorch with Gnina we can now quickly develop new docking models and pipelines. In the future, we plan to add support for non-grid models such as Graph Neural Networks [8]. This development would allow direct comparison between CNN and GNN scoring functions with identical sampling strategies. We also plan to integrate newly developed deep neural network methods for sampling to replace or augment the Monte Carlo sampling currently provided in Gnina [7, 16]. These new sampling methods would provide an opportunity for improving binding site detection for whole protein docking, reducing the computational cost of sampling, and allowing for accurate docking to *apo* protein structures.

## Availability and requirements

Project name: Gnina

Project home page: https://github.com/gnina/gnina

Operating systems: Linux (Docker container available)

Programming language: C++, CUDA

Other requirements: CUDA, Open Babel 3

License: GPL2/Apache License

Any restrictions to use by non-academics: None

## Supplementary Information

Supplementary material 1.

## Data Availability

No datasets were generated or analysed during the current study.
