# Supplementary material for: GNINA 1.3: the next increment in molecular docking with deep learning
Source: J Cheminform. 2025 Mar 2;17:28. doi: 10.1186/s13321-025-00973-x (PMC11874439; doi:10.1186/s13321-025-00973-x)
Supplement: Supplementary file 1 — Supplementary material 1. [file 13321_2025_973_MOESM1_ESM.pdf]

Supporting Information: GNINA 1.3: The next increment in molecular docking with deep learning

Andrew T. McNutt,† Yanjing Li,‡ Rocco Meli,¶,§ Rishal Aggarwal,† and David Ryan Koes*,†

†*Computational and Systems Biology Dept., University of Pittsburgh*

‡*Computational Biology Department, Carnegie Mellon University*

¶*Department of Biochemistry, University of Oxford, Oxford, OX1 3QU, United Kingdom*

§*Present address: Swiss National Supercomputing Center (CSCS), ETH Zurich, 6900 Lugano, Switzerland*

E-mail: dkoes@pitt.edu

Crossdocked2020/Redocked2020 v1.3 Statistics

Table S1: Composition of the updated Crossdocked2020 and Redocked2020 datasets

data set	pockets	poses	ligands	affinity data %
ReDocked2020 v1.3	2900	769794	13846	33.3
CrossDocked2020 v1.3	2900	22566449	13840	41.1

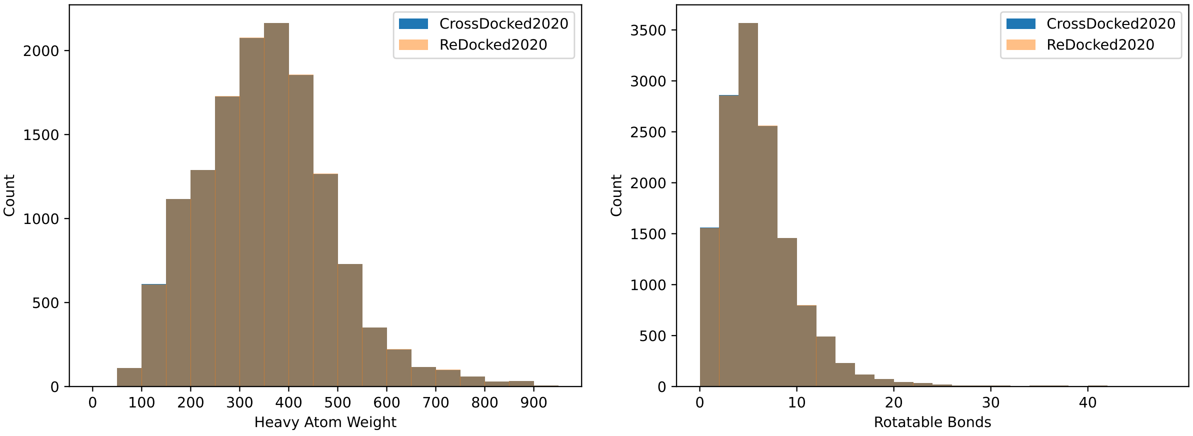

(a) (b)

Figure S1: (a) Heavy atom molecular weight and (b) Number of rotatable bonds of the unique ligands in the v1.3 CrossDocked2020 and Redocked2020 datasets

Model Training Hyperparameters

The CUDA-accelerated libmolgrid library[1] is used to voxelize atomic three-dimensional coordinates into grids, which are then given to the model as inputs. The grid is composed of 14 atom-type channels for the protein and 14 for the ligand. The atom-types are generally elements and additionally split up oxygen/nitrogen hydrogen bond donors/acceptors and aliphatic/aromatic carbons into their own types. The cubic grid is centered on the ligand with a side length of 23.5 Å and a resolution of 0.5 Å. Retrained models utilize the same training setup as the original models in Francoeur et al.[2]. For KD, the batch size is 50 and the starting learning rate is 0.001. We use a 'clip_value' of 10 to avoid gradient explosion. To augment the data to avoid overfitting, learn an approximation of SE(3) invariance, and reduce generalization error, the center of the input protein-ligand grid is randomly translated up to 6 Å from the ligand's center of mass and randomly rotated. SGD is used as the optimizer with a momentum of 0.9 and weight decay of 0.001. To better train the model, we utilize a step-decreasing learning rate strategy, as in Francoeur et al.[2]. We evaluate the error on a subsample of the training dataset whose size is proportional to the training dataset size every 1000 iterations. If the error doesn't decrease for

‘step_when’ times, the learning rate will be divided by 10. After decreasing the learning rate for ‘step_end_cnt’, the training terminates. The proportion of reduced dataset size to the full training dataset size and values of ‘step_when’ and ‘step_end_cnt’ are shown in Table S2.

The PyTorch KD training scripts are available here: `https://github.com/YanjingLiLi/GNINA_Knowledge_Distillation`.

Table S2: Training hyperparameters used for the baseline models and Knowledge Distilled models

	percent reduced	step_when	step_end_cnt
General dataset	100	88	4
Redock dataset	36.3	200	3
Crossdock dataset	0.886	200	3

Table S3: Ensemble KD teacher models, student architecture, and distillation dataset.

Teacher	Student Architecture	KD Dataset
`general_default2018` ensemble	Default2018	PDBBind General
`crossdock_default2018` ensemble	Default2018	CrossDock2020 v1.3
`redock_default2018` ensemble	Default2018	ReDock v1.3
All Default2018 Ensemble	Default2018	CrossDock2020 v1.3
`dense` ensemble	Dense	CrossDock2020 v1.3
`dense` ensemble	Default2018	CrossDock2020 v1.3

Table S4: Pose prediction evaluation dataset statistics

dataset	task	protein-ligand pairs	pockets
[3]	cross-docking	7970	92
Posebusters benchmark set	redocking	308	
Astex diverse set	redocking	85	

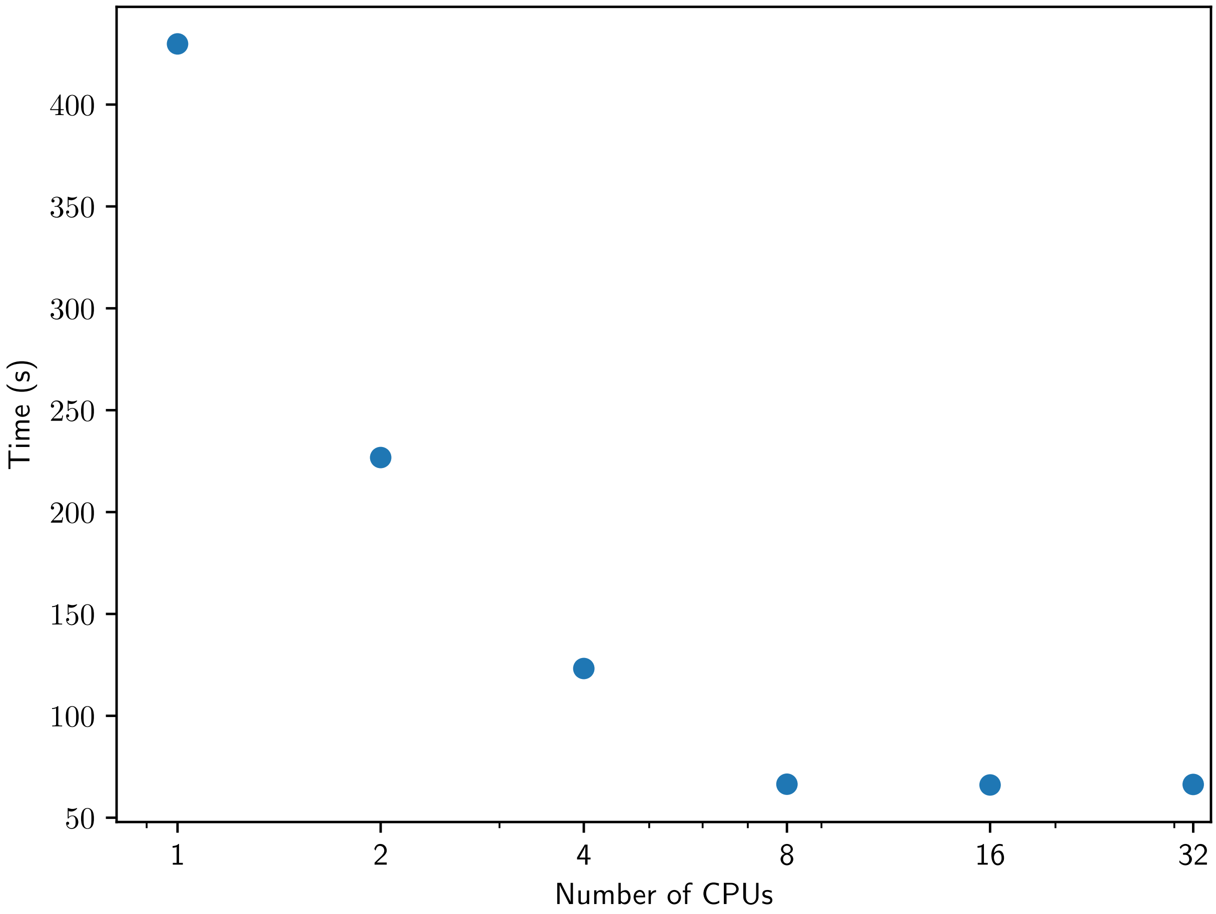

Figure S2: For a single system (PDB ID: 3PRS), we evaluate the time to complete docking with varying number of CPUs and no GPU. The docking time decreases as the number of CPUs increases. When the number of CPUs is greater than 8, the docking time does not decrease further. This plateau in docking time is due to the default number of monte-carlo chains, 8, where each CPU is assigned to a different monte-carlo chain.

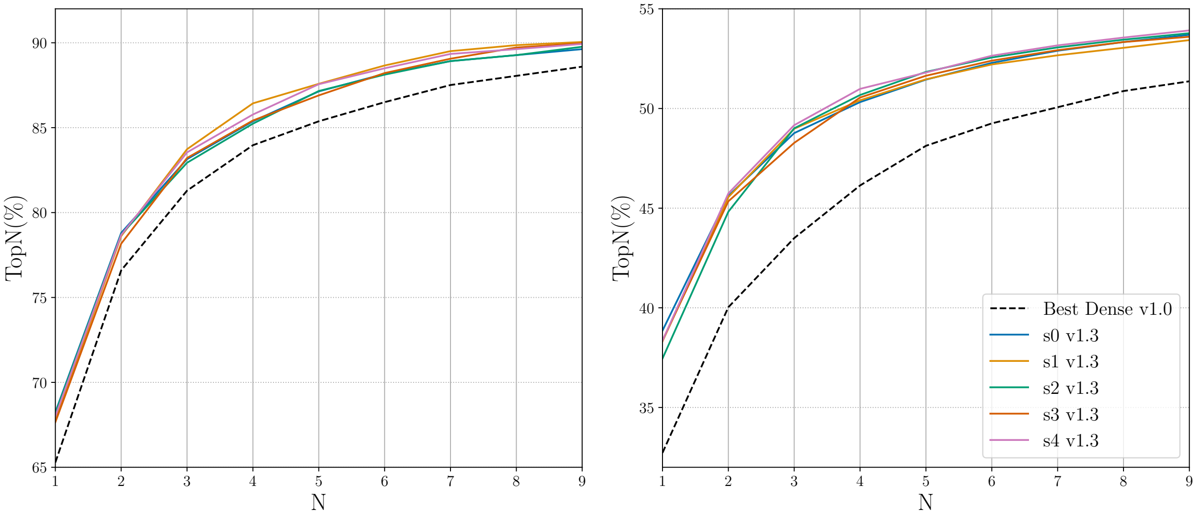

(a) Redocking (b) Crossdocking

Figure S3: Comparing the pose ranking performance via TopN of the dense models trained on the updated CrossDock2020 v1.3 dataset to the best performing model trained on the CrossDock2020 v1.0 dataset. All of the retrained dense models rank poses better for both redocking and cross-docking tasks.

Benchmarking Gnina Docking

We calculate the average docking time per protein-ligand system, "Avg Time per System" on a random 100 complex subset of the PDBbind v2016 core set.[7] We utilize the hyperfine[8] benchmarking tool to measure the docking runtime. This performs a minimum of five docking runs for each system, running additional dockings in cases exhibiting high variance. The mean time per protein-ligand complex computed by hyperfine is averaged over the 100 complexes to compute the "Avg Time per System". Timing measurements were carried out on a 4-core 4.00GHz Intel i7-4790K processor, equipped with 32 GB of RAM and a 10 GB RTX 3080 graphics card utilizing CUDA 12.2 and cuDNN 7.0.5 software packages. Benchmarkings were executed on an otherwise idle system, allocating four processing cores (`cpu=4`) for docking. Timings indicating "CPU-only" utilized the command line flag: (`--no-gpu`), which ignores the GPU during docking.

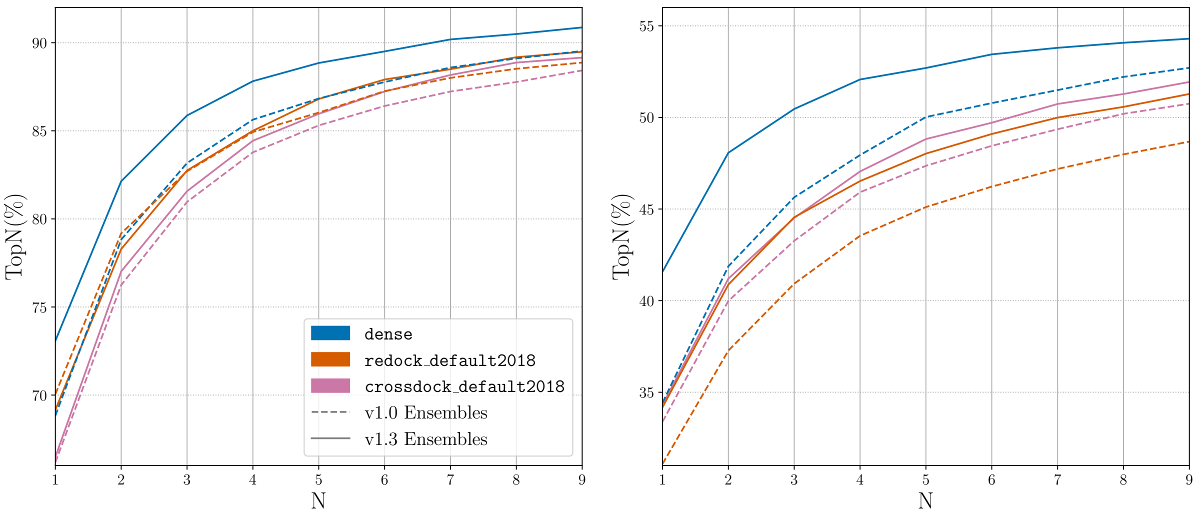

(a) Redocking (b) Cross-docking

Figure S4: Comparing the pose ranking performance via TopN of the basic ensembles, ensembles composed of models only differing in their initialization, trained on the updated CrossDock2020 v1.3 dataset to the same ensemble trained on the old CrossDock2020 v1.0 dataset. All retrained ensembles rank poses more accurately for cross-docking, however, the `redock_default2018` ensemble performs about the same on redocking.

PDB IDs for the benchmark

2YFE, 3P5O, 3UEV, 4W9L, 1QF1, 4AGQ, 3UDH, 3ARQ, 1BZC, 3AG9, 4TMN, 2VW5, 3ZDG, 1H22, 4GR0, 1O0H, 4IVB, 3KWA, 2XDL, 4F3C, 1NC3, 4GKM, 3DD0, 4JSZ, 1MQ6, 3B68, 4EO8, 3BGZ, 2YGE, 4DDH, 2QE4, 4DE2, 1GPK, 1UTO, 4AGN, 2R9W, 2WVT, 4EA2, 4E6Q, 2C3I, 3GY4, 3G31, 2P4Y, 4J28, 3U5J, 5A7B, 3B65, 4OGJ, 1Z6E, 2ZCR, 3UEU, 4WIV, 2VVN, 4LLX, 3UUO, 2XNB, 3B5R, 4ABG, 2V00, 5ABA, 3G2Z, 2WEG, 3O9I, 4IVD, 3ZSO, 1S38, 3GBB, 4LZS, 4QAC, 1U1B, 4W9C, 1Z95, 4E5W, 1H23, 4F9W, 3UI7, 4DJV, 3NX7, 4DDK, 3QGY, 2YMD, 4JFS, 4TWP, 5TMN, 2WCA, 3EJR, 2ZB1, 4KZQ, 4F09, 2WN9, 3RYJ, 3PRS, 3B27, 4OWM, 4JXS, 2WTV, 3JYA, 4DE1, 3OE4, 1NVQ

Table S5: Average redocking performance of GNINA scoring functions. Redocking performance evaluated on the PDBbind v2019 refined dataset.[4,5] ± indicates the standard deviation of five models trained from different random seeds, except in the case of "All Default2018" distillation which only has three student models.

	Single Model	Ensemble	KD → Default2018	KD → Dense
Model name	Top1 (%)			
crossdock_default2018	65.01(±0.62)	66.48	66.33(±0.45)	N/A
redock_default2018	63.23(±0.69)	66.41	65.04(±0.43)	N/A
general_default2018	66.82(±0.71)	66.41	65.92(±0.48)	N/A
dense	67.97(±0.25)	73.08	65.69(±0.44)	69.71(±0.48)
All Default2018	N/A	70.61	69.46(±0.18)	N/A

Table S6: Average crossdocking performance of GNINA scoring functions. Crossdocking performance is evaluated on the Wierbowski et al.[3] dataset. ± indicates the standard deviation of five models trained from different random seeds, except in the case of "All Default2018" distillation which only has three student models.

	Single Model	Ensemble	KD → Default2018	KD → Dense
Model name	Top1 (%)			
crossdock_default2018	33.43(±0.59)	34.30	33.07(±0.22)	N/A
redock_default2018	31.08(±0.78)	32.87	32.22(±0.33)	N/A
general_default2018	33.23(±0.69)	35.43	34.17(±0.48)	N/A
dense	38.27(±0.51)	41.57	35.93(±0.20)	38.38(±0.39)
All Default2018	N/A	36.99	35.93(±0.40)	N/A

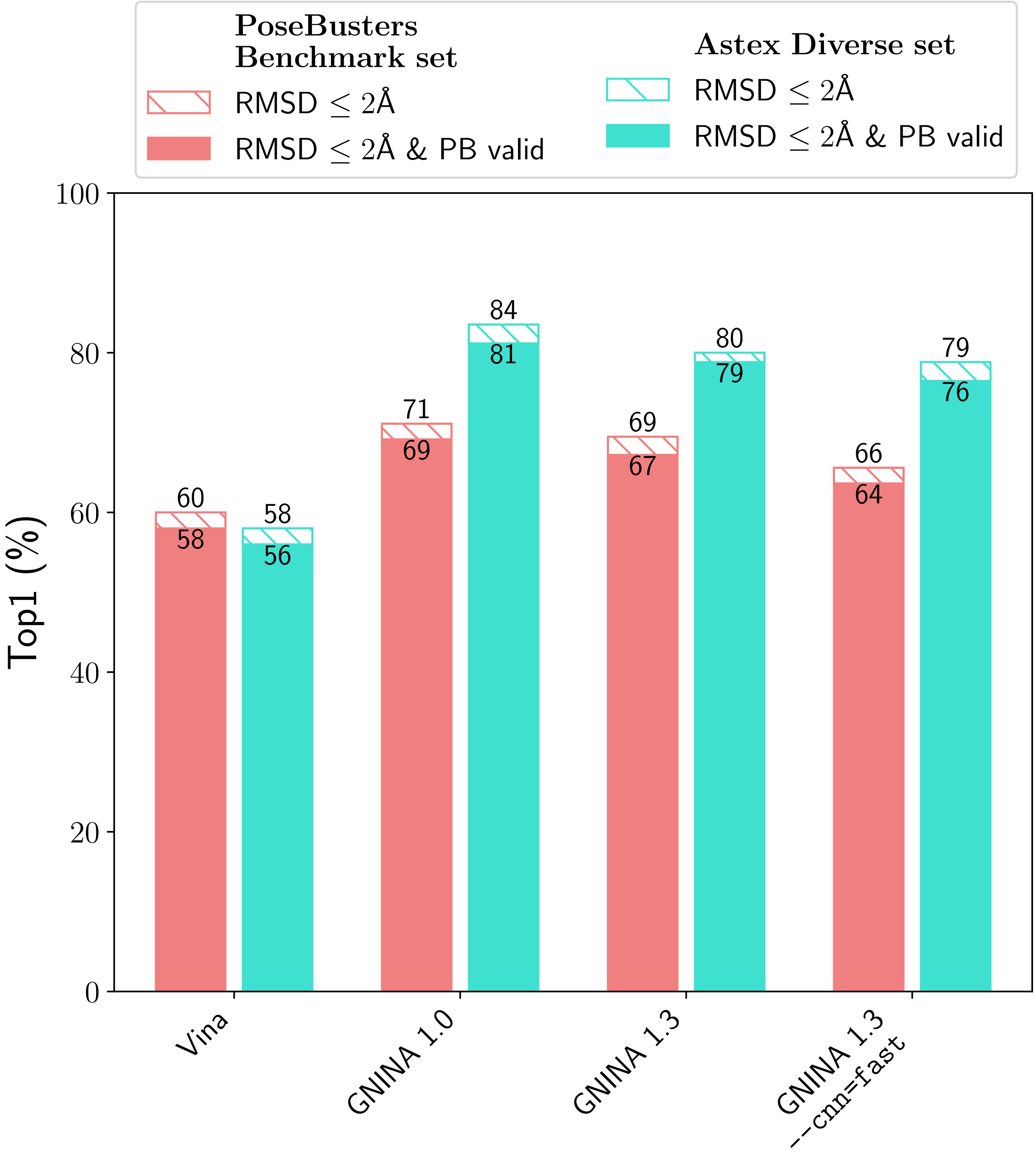

Figure S5: Redocking Top1 performance of the GNINA scoring functions on the Posebusters and Astex Diverse datasets. The hatched bars indicate all Top1 poses that are ≤ 2ÅRMSD from their ground truth pose, while the solid bar additionally filters the poses using the Posbusters' plausibility checks.[6]

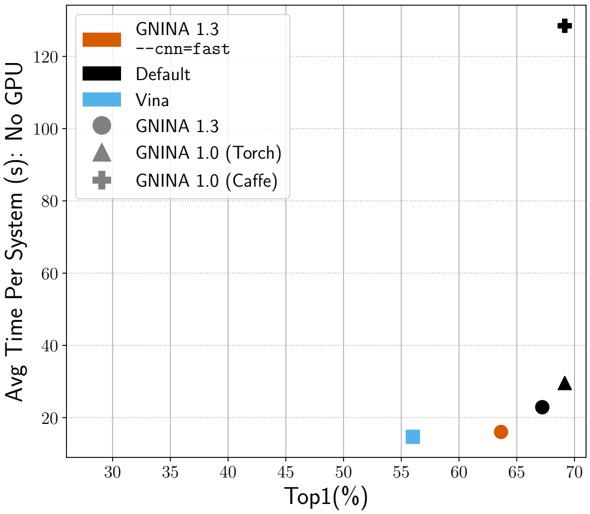

(a) No GPU: Redocking

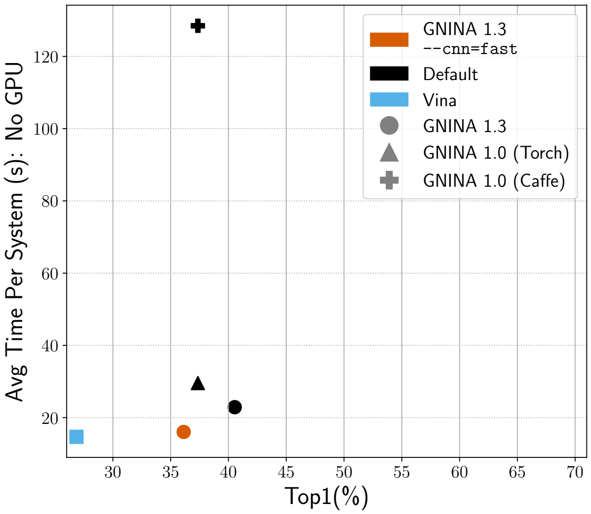

(b) No GPU: Crossdocking

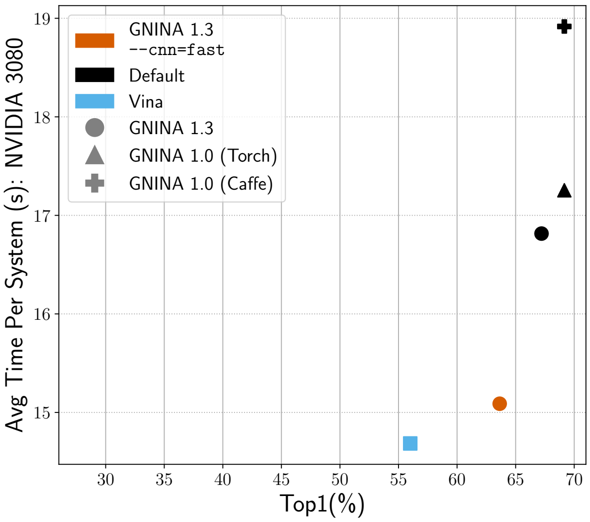

(c) GPU: Redocking

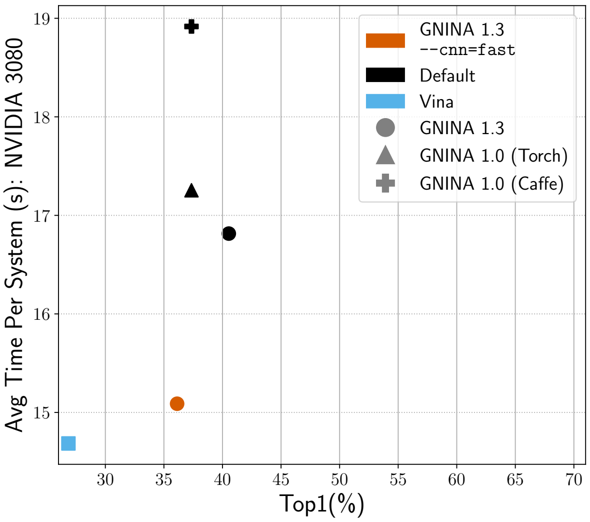

(d) GPU: Crossdocking

Figure S6: Comparing the time to dock a protein-ligand pair to redocking (left column) and crossdocking (right column) performance. Top row provides the average time to dock when no GPU is used during docking and the bottom row provides the average time to dock when a NVIDIA RTX 3080 GPU is used during docking. Redocking performance is measured by the Posebusters benchmark dataset[6] and crossdocking performance is measured by the Wierbowski et al.[3] crossdocking dataset. Moving from Caffe to Torch (+ to ▲) as the deep learning platform significantly reduces the computational cost of docking both with and without a GPU. The GNINA 1.3 Default Ensemble (●) shows higher performance on the crossdocking task than the 1.0 Default Ensemble with a reduced computational cost. GNINA 1.3 Default Ensemble (●) shows slightly reduced performance on redocking compared to the 1.0 Default Ensemble. The fast model (●) was selected to be at the pareto-frontier of docking speed and pose selection performance for both redocking and crossdocking tasks, it shows significant pose selection performance boost over Vina without adding much computaional cost.

Table S7: Evaluating the computational cost and docking performance of the CNN scoring functions when used to rescore and rank the output poses. Autodock Vina is used as a baseline since using no CNNs in Gnina is the same as Vina. The **bold numbers** indicate the fastest time and highest docking performance.

Model	GPU Time (s)	CPU-only Time (s)	Redock Top1 (%)	Crossdock Top1 (%)
Vina	14.7	14.7	56.00	26.85
GNINA 1.0 (Caffe)	18.9	128.5	**69.16**	37.35
GNINA 1.0 (PyTorch)	17.3	29.6	**69.16**	37.35
GNINA 1.3	16.8	22.9	67.21	**40.54**
GNINA 1.3 `--cnn=fast`	**15.1**	**16.0**	63.64	36.13

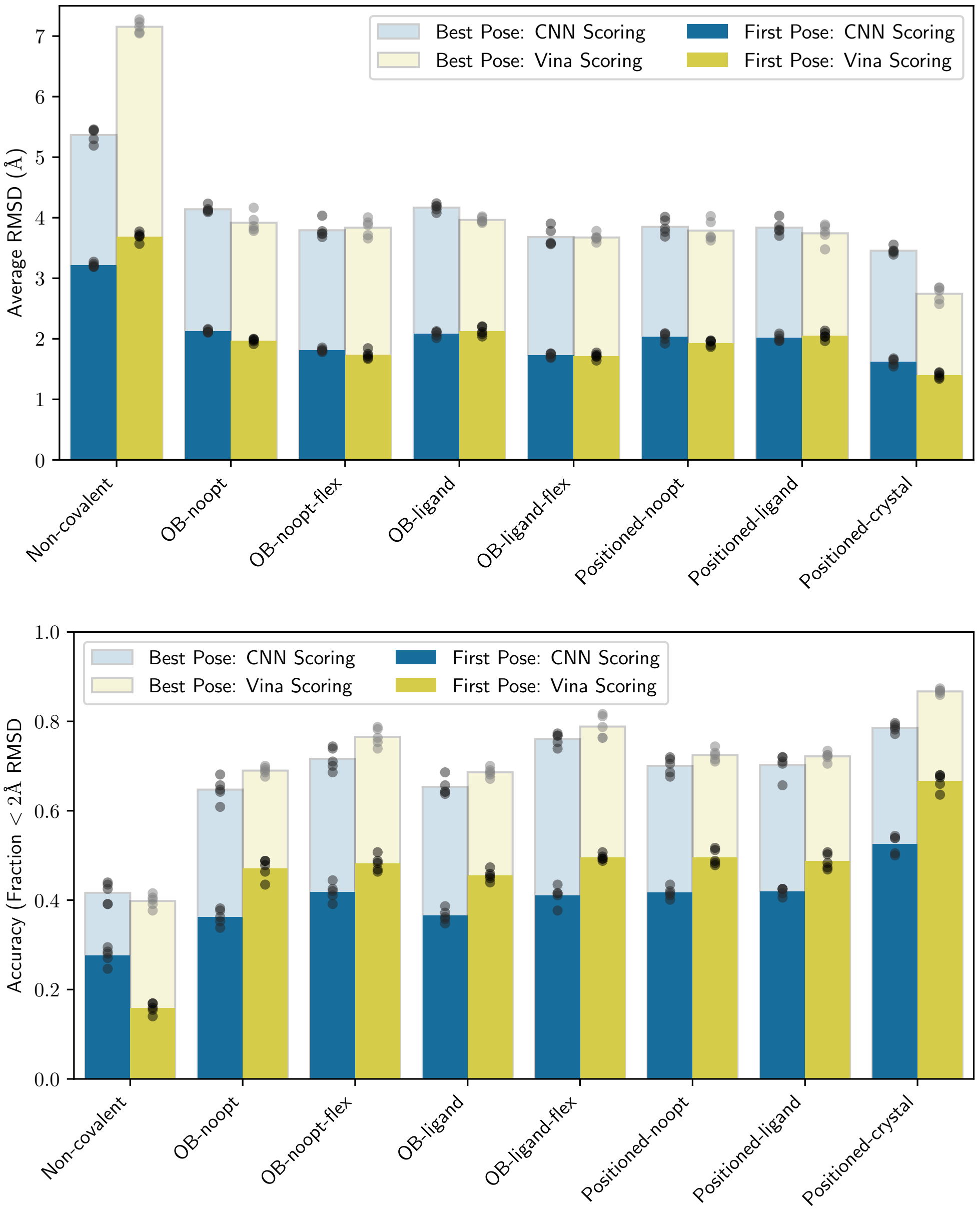

Figure S7: Comparison of average RMSD (top) and accuracy (bottom) for different covalent docking settings on the Scarpino et al.[9] benchmark. Circles are results of one of five different random seeds and indicate the amount of variation. Non-covalent: no covalent bonding is specified, regular docking. OB-nopt: the default, OpenBabel heuristics are used to determine positioning of covalent bond. OB-noopt-flex: same as OB-noopt, but side-chain of covalent residue on protein is treated flexibly. OB-ligand: same as OB-noopt, but covalently bound ligand geometry (including covalent bond) is optimized with the UFF force field. OB-ligand-flex: same as OB-ligand with flexible receptor side-chain. Positioned-nopt: position of covalent ligand atom is manually specified to the correct position from the experimental structure instead of calculated using OpenBabel's heuristics. Positioned-ligand: similarly only with UFF minimization of covalently bound ligand. Positioned-crystal: same as Positioned-noopt, but the ligand conformer used is taken directly from the experimental structure instead of generated from SMILES.

References

(1) Sunseri, J.; Koes, D. R. Libmolgrid: graphics processing unit accelerated molecular gridding for deep learning applications. *Journal of chemical information and modeling* **2020**, *60*, 1079–1084.

(2) Francoeur, P. G.; Masuda, T.; Sunseri, J.; Jia, A.; Iovanisci, R. B.; Snyder, I.; Koes, D. R. Three-Dimensional Convolutional Neural Networks and a Cross-Docked Data Set for Structure-Based Drug Design. *Journal of Chemical Information and Modeling* **2020**, *60*, 4200–4215.

(3) Wierbowski, S. D.; Wingert, B. M.; Zheng, J.; Camacho, C. J. Cross-docking benchmark for automated pose and ranking prediction of ligand binding. *Protein Science* **2020**, *29*, 298–305.

(4) Wang, R.; Fang, X.; Lu, Y.; Wang, S. The PDBbind database: Collection of binding affinities for protein- ligand complexes with known three-dimensional structures. *Journal of medicinal chemistry* **2004**, *47*, 2977–2980.

(5) Liu, Z.; Su, M.; Han, L.; Liu, J.; Yang, Q.; Li, Y.; Wang, R. Forging the basis for developing protein–ligand interaction scoring functions. *Accounts of chemical research* **2017**, *50*, 302–309.

(6) Buttenschoen, M.; Morris, G. M.; Deane, C. M. PoseBusters: AI-based docking methods fail to generate physically valid poses or generalise to novel sequences. *Chemical Science* **2024**,

(7) Su, M.; Yang, Q.; Du, Y.; Feng, G.; Liu, Z.; Li, Y.; Wang, R. Comparative assessment of scoring functions: the CASF-2016 update. *Journal of chemical information and modeling* **2018**, *59*, 895–913.

(8) Peter, D. hyperfine. `https://github.com/sharkdp/hyperfine`, 2020.

(9) Scarpino, A.; Ferenczy, G. G.; Keserű, G. M. Comparative evaluation of covalent docking tools. *Journal of Chemical Information and Modeling* **2018**, *58*, 1441–1458.
